# The Role of Nurse on the Treatment Decision Support for Older People with Cancer: A Systematic Review

**DOI:** 10.3390/healthcare11040546

**Published:** 2023-02-12

**Authors:** Hiroko Komatsu, Yasuhiro Komatsu

**Affiliations:** 1 Japanese Red Cross Kyushu International College of Nursing, 1-1 Asty, Munakata-City 811-4157, Fukuoka, Japan; 2 Department of Healthcare Quality and Safety, Gunma University Graduate School of Medicine, 3-39-22 Showa-machi, Maebashi 371-8511, Gunma, Japan

**Keywords:** older adults, cancer, decision-making, nurse

## Abstract

**Background**: The number of older adults with cancer is increasing worldwide. The role of nurses in supporting patients’ decision-making is expanding, as this process is fraught with complexity and uncertainty due to comorbidities, frailty, cognitive decline, etc., in older adults with cancer. The aim of this review was to examine the contemporary roles of oncology nurses in the treatment decision-making process in older adults with cancer. **Methods**: A systematic review of PubMed, CINAHL, and PsycINFO databases was conducted in accordance with PRISMA guidelines. **Results**: Of the 3029 articles screened, 56 full texts were assessed for eligibility, and 13 were included in the review. We identified three themes regarding nurses’ roles in the decision-making process for older adults with cancer: accurate geriatric assessments, provision of available information, and advocacy. Nurses conduct geriatric assessments to identify geriatric syndromes, provide appropriate information, elicit patient preferences, and communicate efficiently with patients and caregivers, promoting physicians. Time constraints were cited as a barrier to fulfilling nurses’ roles. **Conclusions**: The role of nurses is to elicit patients’ broader health and social care needs to facilitate patient-centered decision-making, respecting their preferences and values. Further research focusing on the role of nurses that considers diverse cancer types and healthcare systems is needed.

## 1. Introduction

Population aging has substantially contributed to an increasing number of new cancer cases worldwide [1]. The global cancer burden is expected to be 28.4 million cases in 2040, a 47% rise from 2020 [2]. The number of new cancer cases among older adults (aged 65 years and older) is expected to double by 2035 (14 million) [1]. Age is a risk factor for cancer due to the duration of carcinogenesis, the vulnerability of aging tissues to environmental carcinogens, and other bodily changes that favor the development and the growth of cancer [3].

Healthcare providers (HCPs) involved in the treatment of older adults with cancer face many challenges. Older adults with cancer often have age-related frailty [4,5], comorbidities [6,7], and polypharmacy [8,9], which complicate the cancer diagnosis and create uncertainty in decisions about treatment goals and outcomes [7]. In addition, the involvement of caregivers and other key persons in decision-making affects the decision structure and process [10,11]. Thus, clinical practice guidelines for older patients with cancer provide recommendations for the appropriate implementation of validated and standardized clinical assessment tools and decision-making models for this vulnerable and prevalent demographic group [12]. However, over 50% of older patients with advanced cancer experience severe toxicity during the first 3 months of chemotherapy [13]. In managing cancer drug-related adverse effects and the quality of life, assessment of the values and preferences of older adults with cancer is critical to informed treatment decision-making [14].

In recent years, there has been growing evidence that geriatric assessments (GAs) can be used to assess and manage the vulnerability of older adults with cancer [7,14], and can aid in shared decision-making (SDM) regarding treatment and interventions among patients, caregivers, and oncologists [12]. Nurses are at the frontline in the care of patients with cancer, particularly in this new era of SDM [15]. Advanced nurse practitioners play a pivotal role in determining and facilitating the preferences of patients with cancer [16]. The nursing role during cancer SDM can be complicated and requires flexibility [17]. Although the importance of nurses’ roles has been discussed, a synthesis of the roles of nurses in the treatment decision-making process of older adults with cancer and their effects is lacking. Therefore, this systematic review examined the contemporary roles of oncology nurses throughout the cancer treatment decision-making process of older adults with cancer.

## 2. Methods

### 2.1. Search Question

What roles do oncology nurses play in the treatment decision-making process of older adults with cancer?

### 2.2. Search Strategy

This review was based on a systematic, comprehensive search of three databases, including CINAHL, PubMed (via MEDLINE), and PsycINFO, and was conducted in accordance with PRISMA guidelines [18]. Manual searches of reference lists and gray literature were also performed to identify relevant articles. Searches were limited to articles published in English, database inception to September 2022. To address the research question, a broad range of key search terms based on the MeSH (Medical Subject Headings) topics of “decision making”, “older adults”, “cancer”, and “nurse” were used. For other MESH terms, and a combination of free-text searches refer to Supplementary Files S1 and File S2.

### 2.3. Eligibility Criteria

The literature searches aimed to identify qualitative, quantitative, and mixed-method studies that provided a description of the roles of the nurse throughout the treatment decision-making process for older adults with cancer. Studies were limited to those that focused on adults ≥ 60 years of age. Additionally, reviews, letters, case studies, editorials, and conference abstracts were excluded.

### 2.4. Quality Appraisal

Two reviewers (H.K. and Y.K.) discussed and selected the articles to be included in this review. Studies were selected using a two-step process. Articles were first screened by title and abstract to determine their relevance to the search question. The PRISMA search strategy [18] was used to filter articles and remove duplicates. Full-text articles were then retrieved and independently reviewed to determine whether the inclusion criteria were met. Two researchers (H.K. and Y.K.) independently evaluated the studies that met the inclusion criteria for methodological quality using the Mixed Methods Appraisal Tool (MMAT), V.2018 [19].

### 2.5. Thematic Analysis

We provided a narrative summary by conducting a qualitative synthesis to identify key themes based on thematic analysis [20]. First, free line-by-line coding of findings from included studies was conducted into related field. Next, thematic analysis was undertaken to construct themes related to the research questions across studies.

## 3. Results

**Figure 1:**
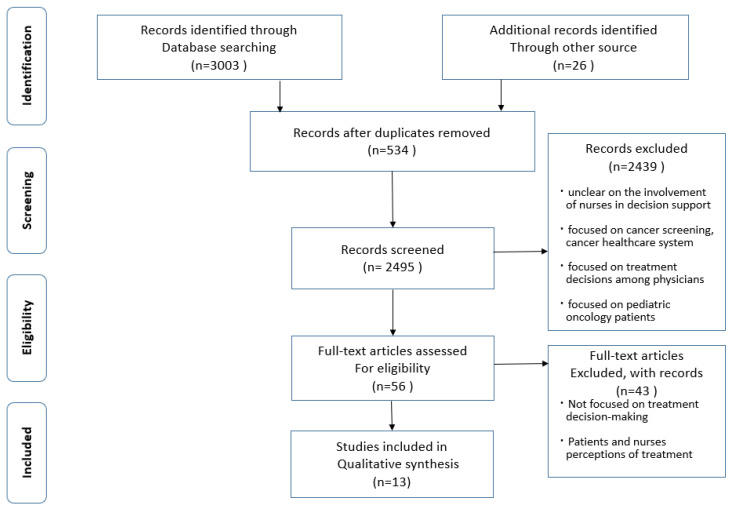
PRISMA flow diagram.

A total of 3029 articles were identified through database searches supplemented by manual searches. Of these, 534 duplicates were removed; studies that were unclear on the involvement of nurses in decision support or did not focus on decision support in patients with cancer, such as those focused on cancer screening, cancer healthcare system, and treatment decisions among physicians, were excluded. Studies that focused on pediatric oncology patients were also excluded because they did not meet inclusion criteria. The remaining articles underwent full-text review and 13 were deemed suitable for inclusion (Figure 1).

### 3.1. Study Characteristics

Table 1 presents the main characteristics of the 13 studies included in this review. Seven studies were conducted in European countries [21,22,23,24,25,26,27], and six in the USA or Canada [28,29,30,31,32,33]. Two studies used a quantitative cross-sectional design [22,24], one used a retrospective cohort design [26], one used a quasi-experimental design (pre-post study design) [32], one used a mixed-method design [28], six used a qualitative design [21,23,25,27,30,31], and two were case studies [29,33]. Only one study examined the effect of a nurse-led GA on treatment modifications and outcomes [26]. One pre-post study examined the effect of a Communication Skills Training module on the HCP’s SDM approach to meetings with older adults with cancer and their family [32]. Two case studies described the usefulness of nursing practices in the treatment decision-making of older adults with cancer [29,33]. One quantitative cross-sectional study examined the perception of HCPs (including nurses) on treatment decisions of older adults with cancer [24]. One cross-sectional questionnaire survey investigated older women’s preferences for receiving information about breast cancer treatment options [22]. Qualitative studies focused on perceptions in older adults with cancer and their partners’ decision-making [30], and the perceptions of older adults with cancer [31], HCPs [21,27], and older adults with cancer, their families, and HCPs [23,25].

### 3.2. Quality Assessment

Among the 13 included studies, two were case studies and did not undergo quality assessment; the remaining 11 primary studies underwent methodological quality assessment using the MMAT [19]. These studies met 100% of the quality criteria, with the exception of one study that met 75% of the quality criteria, and had high quality scores (Table 1, Supplementary File S3).

### 3.3. Themes of Included Studies

The data were categorized into three themes regarding the nurse’s role in the treatment decision-making process of older adults with cancer: (a) accurate GAs, (b) provision of available information, and (c) advocacy.

### 3.4. Accurate GAs

The oncology nurse plays an important role in assessing the factors to be considered in the cancer treatment decision-making process by properly implementing GAs in older adults with cancer. Festen et al. conducted a retrospective analysis of the outcomes of nurse-led GAs and patient preference assessment; they found that nurse-led GAs may lead to the tailoring of treatment decisions to the patient’s frailty status and preferences, and improve outcomes [26]. There was no significant difference in one-year mortality between the unchanged and modified group (29.7% versus 26.1%, *p* = 0.7). There were, however, significantly fewer days spent in hospital (median 5 vs. 8.5 days *p* = 0.02) and fewer grade II or higher postoperative complications (13.3% versus 35.5% *p* = 0.005) in the modified group. Additionally, two case studies reported on the usefulness of advanced practice nurses. Specifically, Shahrokni et al. reported on comprehensive geriatric evaluations and effective GA-based interventions performed at the Geriatrics Service department in the Memorial Sloan Kettering Cancer Center [29]. At this center, geriatric nurse practitioners performed GAs to identify geriatric syndromes, derive patient references, and efficiently communicate with patients, caregivers, oncologists, and primary care physicians [29]. Similarly, Strohschein et al. conducted a case study of an 89-year-old man with head and neck cancer [33]. The authors concluded that oncology nurses could identify and address age-related concerns, facilitate communication, and contribute to personalized care by integrating GA tools into their practice.

In these three studies, nurses were responsible for comprehensive GAs in collaboration with a multidisciplinary team for cancer treatment [26,29,33]. Nurses conducted adequate comprehensive GAs by selecting standardized assessment tools for each domain, based on the geriatric domain framework. GAs performed by nurses led to timely interventions, proactive follow-ups, support of patient goals and values, and coordination of care. However, as GAs aim to tailor care to individual patients and improve outcomes [26], extra time must be spent on patient assessments during the decision-making process [26,33]. Thus, time is sometimes a limiting factor in the implementation of GAs.

In older people, oncology nurses can facilitate treatment planning and recovery by conducting an accurate GA. A key issue related to this is the acquisition of competencies for effectively and efficiently assessing patients in the presence of time constraints.

### 3.5. Provision of Available Information

Nurses play a role in the timely provision and sharing of information in the treatment decision-making process, based on a relationship of trust with the patient. A qualitative study reported that nurses attempted to compensate physicians’ shortcomings by providing patients with additional information and opportunities for discussion, and sought to form trusting relationships to enable a continuity of care and facilitate access to support during treatment [21]. As older adults are sometimes reluctant to share personal information, nurses should focus on building trusting relationships with elderly patients [21]. Furthermore, pertinent patient information is not always available at the time of treatment decisions. Therefore, nurses need to continuously collect quality, available, and timely information about older adult patients, assessing what is happening in their daily lives, to enable informed treatment decisions [21].

The importance of nurses’ provision of information was also indicated in studies on the perceptions of older adults with cancer. In a survey by Burton et al. of older adults with breast cancer who needed information for treatment decision-making, nearly 40% indicated that a face-to-face discussion with a nurse was their preferred source of information [22]. Furthermore, most women stated that a breast care nurse (45/55, 82%) was the ideal person with whom they would discuss their treatment decisions [22]. These results suggest the importance of the role of nurses in providing information and ensuring that women receive their preferred level and amount of information, as well as their involvement in treatment decision-making using decision support tools.

On the other hand, in a qualitative study on perceptions regarding treatment decisions in older adults with cancer, the majority of patients were satisfied with the communication with their oncologists, and none of the patients mentioned nurses as having input or providing support in their treatment decision-making process [31]. Therefore, nurses must be actively involved in decision-making processes so that their role is recognized by patients. For example, nurses may coach patients on how to seek evidence-based discussions regarding treatment options and provide supplementary education on treatment options.

McWilliams et al. conducted a qualitative study on treatment decision-making in older adults with cancer and dementia and their families, as well as HCPs, including specialized nurses [23]. One important theme was the effective communication of clinically relevant information, and the authors provided the following recommendations: taking more time with the patient, exchanging information, and understanding the options for cancer treatment. HCPs may need to speak slowly and repeat information several times to help patients and their families navigate treatment decision-making, and avoid vague descriptions of side effects, complex information, and a lack of timely information [23]. Shahrokni et.al points out that effective and efficient communication between oncologists and primary physicians or geriatricians, and nurses, especially among older people and their families, needs to be promoted to drive decision-making among older people [29].

Training in communication skills is required to promote the communication of clinically relevant information. Shen et al. evaluated a Communication Skills Training module for HCPs by applying a SDM approach to meetings with older adults with cancer and their family [32]. The results indicated a significant effect of training on overall skill; HCPs’ self-efficacy in utilizing communication skills related to shared geriatric decision-making significantly increased from pre- to post-training. Communicating in a way that promotes true SDM is even more important when facing critical treatment decisions in older adults with cancer who may experience cognitive decline [32].

Studies on the perceptions of HCPs show that nurses who are trusted by patients play a role in treatment planning through the timely provision of information. Survey studies of the perceptions of older people with cancer highlighted the importance of nurses providing information, while other studies showed that there was little recognition of the input or support provided by nurses during treatment planning. Therefore, nurses need to be actively involved in the decision-making process to make patients aware of their role and to strengthen and train their communication skills.

### 3.6. Advocacy

Oncology nurses play an important role in advocating respect for individual values and preferences of older adults with cancer in their treatment decisions. Bridges et al. surveyed clinicians, including nurses, on the characteristics of cancer treatment decision-making in older patients with cancer and found that nurses play an important role in advocating for the patient’s autonomy and the right to make informed decisions [21]. Oncology nurses involved in multidisciplinary teams focus on complex patient-centered information, such as comorbidities, psychosocial and supportive care needs, and patient preferences, indicating the importance of nurses’ input in calling attention to broader issues at the meeting [21]. However, there is a difficulty in nurses providing consistent contributions to multidisciplinary team meetings [21].

On the other hand, Tariman et al. [28] reported on the preferences of older adult patients newly diagnosed with symptomatic myeloma for participation in the decision-making process and found that most patients wanted to share treatment decision-making with their physicians or make decisions themselves. Therefore, physicians and nurse practitioners must practice full disclosure of treatment options to their patients so that they can make truly informed decisions [28]. Further, the authors discussed the importance of the following roles of oncology nurses for respecting and helping individual patients with their preferences: (a) making sure patients receive disease and treatment-related information, (b) encouraging patients to express their decisional role preference to the physician, (c) developing a culture of mutual respect and value of the patient’s desire for autonomy in treatment decision-making, (d) acknowledging that the patient has a right to make treatment choices, and (e) providing psychological support to the patient during decision-making, from the time of diagnosis to the end-of-life. Because the level of preference for participation is highly variable across patients, and may have personal meaning for each patient, physicians and oncology nurses must also elicit the patient’s preferences, explore what participation truly means for him or her, and facilitate the patient’s decision-making process [28].

The utility of decision aids (DAs) in eliciting patient preferences and providing proactive support has been evaluated. In a study of HCPs by de Angst et al. [24], 60% of nurses used DAs to elicit individual patient preferences, suggesting that DAs can be beneficial in supporting SDM. However, oncology nurses were more in favor of DAs than oncologists. In a study of older adults with advanced prostate cancer and their decision partners by Jones et al. [30], participants viewed DAs as helpful in treatment decision-making. DAs allowed issues that they were not aware of to be highlighted, thereby helping them to consider the issues in depth and discuss them with HCPs [30]. Enabling patients and decision partners to discuss issues more thoroughly and providing the time to do so improved their understanding and confidence in their decisions [30]. Additionally, DAs facilitate closer patient–HCP relationships, allowing for more patient-centered and productive conversations [30].

Older adults with cancer often have adult children or spouses involved in treatment decisions [25,27]. Therefore, nurses need to consider the impact of family involvement and family relationships on decision-making processes when supporting the patient’s decision-making. Griffiths et al. indicated the necessity of an assessment that considers multiple factors and ensures psychological well-being in order to help patients apply their individualized abilities in the decision-making process [25]. Dijkman et al. explored how surgeons and nurses perceive the involvement of adult children of older patients with cancer in treatment decision-making [27]. The results indicated that nurses use the following six strategies to support positive family involvement in treatment decision-making: focus on the patient, acknowledge different perspectives, involve adult children, get to know the family system, check that the patient and family members understand the information, and stimulate communication and deliberation with adult children [27]. However, involving families in treatment decision-making also triggers specific complexities and challenges in treatment decision conversations that call for the development and implementation of practical patient- and family-centered strategies [27].

Studies on the perceptions of HCPs demonstrate the need for both nurses and physicians to fully disclose all treatment options to enable patients to make informed decisions. In particular, the preferred level of participation varies greatly from patient to patient and may have personal implications for each patient, and attention should be paid to the influence of family involvement and family relationships on decision making. Nurses need to develop communication skills to support patients’ decision making, by eliciting patients’ information needs and preferred level of participation.

## 4. Discussion

This review is unique in that it focused on the role of nurses in the treatment decisions of older adults with cancer. Previous work reported on physicians’ perceptions of the decision-making process in patients with cancer [34,35,36] or the role of nurses [37]. One of the novel features of this review is the inclusion of data on the effect of GAs by nurses. By conducting GAs, nurses identified geriatric syndromes, elicited patient preferences, and promoted efficient communication with the patients, caregivers, and physicians. The current literature suggest that tailoring treatment decisions to a patient’s frailty status and preferences leads to improvements in patient outcomes.

However, time constraints regarding the implementation of GAs were mentioned [26]. Therefore, for nurses to fulfill their expected role in a multidisciplinary team, they need to acquire competency in efficiently and effectively conducting GAs. The ability of oncology nurses to implement geriatric screening and assessment depends on additional training [33,38], as well as having the time, space, and institutional support to conduct such assessments [39,40]. Outlaw et al. provided an overview of the field of geriatric oncology and highlighted recent breakthroughs in the use of GAs in cancer care [41]. GAs are now recommended for all older adults with a new cancer diagnosis, according to recommendations from the American Society of Clinical Oncology [42], National Comprehensive Cancer Network [43], and International Society of Geriatric Oncology [44]. Further work is needed to better understand and overcome the barriers to the broad implementation and utilization of GAs [41].

Although the level of evidence was low, two case studies [29,33] provided clues regarding the development of GA training programs for nurses that are efficient and effective, as well as personalized implementation of GAs in older adults. Festen et al. showed that incorporating nurse-led GAs in decision-making may improve patient outcomes; however, future studies should use prospective cohorts in diverse cancer populations. Randomized controlled trials are needed to accumulate evidence on the effects of nurse-led GAs in decision-making [26].

Older patients with cancer are often overwhelmed by the complexity and sheer volume of information about cancer diagnosis and treatment, which hinders their access to the information they need [31,45]. The present review clarified that nurses play an important role in identifying the information needs of older patients by assessing each patient’s level of understanding and helping them to understand the information. Many older patients with cancer trust their physicians and are satisfied with their provision of information; however, they also experience poor communication during the treatment decision-making process and beyond [31]. For instance, oncologists’ use of medical jargon, the downplaying of treatment side effects, a lack of sensitivity, and a lack of time spent with patients are some of the issues voiced by patients in this regard [31]. Declining numeracy, lower literacy, and increasing age are associated with the desire to conserve time and energy, which may explain the strong preference for face-to-face conversations using lay language. This preference is of concern, as it may lead to inaccurate risk perceptions. Nurses need to use the teach-back method to confirm the patient’s understanding of the information they receive from physicians [46], provide psychological support [37], elicit and identify individual patient-specific information needs, and facilitate accurate risk perception.

On the other hand, the present review shows that older patients with cancer sometimes do not view nurses as professionals from whom they receive important treatment-related information. Oncology nurses are key players in cancer treatment decision-making; however, they face challenges, including barriers in practice, education, institutional policies, and administration [47]. Nurses need to develop communication skills that can guide patients’ information needs by employing a preemptive and proactive approach that reduces these barriers and raises nurses’ roles as key persons in the care of older patients with cancer. To support the treatment and care decisions for older adults with complex health problems, physicians and nurses must have the communication skills to appropriately respond to complex patient needs through multidisciplinary-team meetings and additional information exchange as well as outside of the conference [21]. Furthermore, we believe that health care providers (HCPs) involved in the multidisciplinary-team need to share treatment and care plans using the Collaborative Care Model to facilitate smooth communication [29].

The practice of SDM is recommended as a standard approach in the decision-making process by policymakers and clinical practice guidelines [48,49]. Implementing a communication training program promotes patient engagement and SDM. The cancer treatment decision-making processes that immediately follow diagnosis occur in a team and can be characterized as medically dominated and narrowly focused on cancer pathology [21]. The importance of knowing about patients’ wider health and social care needs is acknowledged by clinicians; however, they experience difficulty in ensuring that this information is available in time to inform cancer treatment decisions [21]. Thus, nurses must undertake a type of compensatory work to enable patients to engage in treatment decision-making processes and make patient-entered decisions [21]. Further, attention should shift towards exploring decision-making process modifications and providing structural support to ensure that patients with cancer with complex needs receive adequate and timely assessments and access to clinical experts with the capacity to support them in arriving at the best treatment decision [21].

DAs enable patients to fit into the treatment decision process and elicit their values and preferences, leading to proactive support by nurses [24]. A systematic review of the effectiveness of DAs for older adults showed that they improve older adults’ knowledge, increase their risk perception, decrease decisional conflict, and seem to enhance participation in SDM [50]. However, few of the studies included in the present review conducted subgroup analysis in adults with low health literacy or numeracy, low-educated adults, frail patients, or other vulnerable subgroups [50]. When applying DAs to older patients with cancer, nurses need to consider several factors, including multi-morbidities, cognitive impairment, and low health literacy. In addition, more evidence concerning the effects of DAs on decision-making in older patients with cancer is needed.

Older patients with cancer often involve adult children or spouses in treatment decision-making. Family can stimulate deliberation and move the conversation beyond a mere medical perspective by considering relevant aspects of a patient’s life; however, patients may withhold information in the presence of their children, or specific complexities and challenges in treatment decision conversations may be triggered [27]. Thus, nurses should develop practical strategies for triadic conversations related to treatment decision-making based on the core elements of a family system approach and family health conversations [27].

## 5. Limitations

One limitation of the present study is that the evidence reviewed was from a small number of studies, highlighting the need for further research that considers populations with diverse cancer types, characteristics of older adults, and diverse healthcare systems. In addition, the role of nurses may differ depending on their expertise, such as general, oncology, geriatric, and advanced practical nurses. Therefore, it is necessary to promote research that considers these subspecialties. Thematic analysis was conducted in a small number of included studies, making it difficult to extract subthemes. The present review was conducted by repeated exchanges of opinions between two researchers with different specialties (i.e., nurse and physician), from review planning to the literature searches, evaluation, and analysis. Since various professionals are involved in decision-making regarding the treatment of older people, future reviews by a multi-disciplinary expert team with collaboration among various specialties are desirable.

## 6. Conclusions

Cancer treatment decision-making in older patients remains a complex issue. A significant finding from the current literature is that the roles of nurses in the decision-making process of older patients with cancer involve performing an accurate GA, providing available information, and advocating respect for individual values and preferences. The role of nurses is to elicit patients’ wider health and social care needs in complex decision-making processes, respecting individual references and values. However, it may be difficult for older adults and their families to perceive the complementary role of nurses in treatment decision-making, and opportunities for nurses to interact with patients may be missed due to time constraints. Further investigations focusing on the role of nurses that consider diverse cancer types, characteristics of older people, and healthcare systems are needed.

## Figures and Tables

**Table 1:** Studies included in the review.

Author(s) and Year of Publication	Study Population and Setting	Objective(s)	Design	Methods	Summary of Themes	MMAT Score, %
Tariman et al. (2014) [28]	Twenty older adults (60 years of age and older) with symptomatic myeloma diagnosed within the past six months. Recruited from the Seattle Cancer Care Alliance (SCCA) or the Northwestern University Myeloma Program (NUMP), USA.	To examine patient perspectives on their personal and contextual factors relevant to treatment decision-making. The second aim was to describe physician perspectives on the treatment-decision making in older adults diagnosed with symptomatic multiple myeloma.	Quantitative. Cross sectional study. Qualitaive.	Semi-structured face-to-face interviews. Descriptive statistics and Triangulation of Qualitative and Quantitative Data.	(1) Disclosure of treatment options to patients. (2) Encouraging patients to express their decisional role preference to the physician. (3) Developing a culture of mutual respect and value the patient’s desire for autonomy for treatment decision-making. (4) Acknowledging that the right to make a treatment choice belongs to the patient.	100
Bridges et al. (2015) [21]	Healthcare professionals (*n* = 22; *n* = 11 nurse specialists; *n* = 11 physician) Recruited from five English NHS hospital trusts in UK.	To investigate how cancer treatment decisions are formulated for older people with complex health and social care needs and the factors that shape these processes.	Qualitative.	Semi-structured face-to-face interviews. Framework Analysis.	(1) Giving patients quality, availability and timeliness of information and opportunities for discussion. (2) Attention of complex patient-centred information and preference in the meeting. (3) Advocating for the patient’s autonomy and right to make informed decisions. (4) Involved in multidisciplinary teams focus on complex patient-centered information, such as comorbidities, psychosocial and supportive care needs, and patient preferences.	100
Burton et al. (2017) [22]	Women, ≥ 75 years, who had been offered a choice between PET and surgery at diagnosis of breast cancer. (*n* = 101) Recruited from 10 NHS breast units across England and Wales.	To further establish older women’s preferences regarding receiving information about breast cancer treatment options (surgery or PET) and quantify issues raised in the interview study. To quantify women’s preferences regarding the presentation of information and establish their preferred decision-making styles.	Quantitative. Cross sectional study.	Multicentre survey using questionaire. Descriptive statistics.	(1) Ensuring that women receive the preferred level and amount of information as well as involvement when making treatment decisions. (2) Help patients reach their preferred level of information and involvement in decision making using decision support tools.	100
Shahrokni et al. (2017) [29]	An 88-years old patient with colon cancer. Recruited from Cancer Center, USA.	To describe how the Geriatrics Service at Cancer Center approaches an older patient with colon cancer from presentation to the end of life, show the importance of geriatric assessment at the various stages of cancer treatment, and how predictive models are used to tailor the treatment.	Qualitative. Case Study Design.	Retrospective case.	(1) Perform geriatric assessment and identify geriatric syndromes. (2) Manage comorbid conditions that could prevent successful cancer treatment. (3) Effectively and efficiently communicate with patient and caregivers, oncologist, and primary care physician.	-
Jones et al. (2018) [30]	Thirty-five pairs of patients and their decision partners (16 pairs reflected patients with less than 6 months since their diagnosis of metastatic castration-resistant prostate cancer). Recruited from Cancer Center, USA.	To describe and understand the lived experience of patients with advanced prostate cancer and their decision partners who utilized an interactive decision aid to make informed, shared treatment decisions.	Qualitative.	Semi-structured interview. Hermeneutic phenomenological approach.	(1) Facilitating to discuss issues thoroughly between patients and decision partners by using decision aids. (2) Facilitating closer patient-healthcare provider relationships by using decision aids.	100
McWilliams (2018) [23]	Patients with a diagnosis of cancer–dementia (*n* = 10), informal caregivers (*n* = 9), and oncology HCPs (*n* = 12). Recruited from a regional treatment cancer centre, UK.	To explore the cancer-related information needs and decision-making experiences of patients with cancer and comorbid dementia, their informal caregivers, and oncology healthcare professionals.	Qualitative. Cross-sectional study.	Semi-structured face-to-face interviews. Thematic analysis.	(1) Communicating clinically relevant information. (2) Suggesting that dementia-related cognitive and communication impairments influence treatment options in relation to potential side effects and appropriate management. (3) Navigating treatment decision-making information.	100
Sattar et al. (2018) [31]	Ten patients aged ≥ 65 in the curative/palliative setting (presenting with breast, prostate, colorectal, or lung cancer) and who had made a treatment decision in the preceding six months. A Cancer Centre, University Health Network or Health Sciences Centre, Toronto, Ontario, Canada,	To explore the factors that were important for accepting or refusing cancer treatment by older adults undergoing chemotherapy and/or radiation therapy.	Qualitative.	Semi-structured face-to-face interviews. Framework Analysis.	(1) Coaching patients on how to seek evidence-based discussion regarding treatment options. (2) Providing supplementary education on treatment options.	100
de Augst et al. (2019) [24]	Health care providers (*n* = 170), including 82 urologists, 31 oncologists, and 57 oncology nurses. Recruited from participants of meeting of the Netherlands Association for Urology and urologists and oncology nurses by the Netherlands Association for Urology and Dutch National Consultation Oncology Nurses, Netherlands.	To evaluate perspectives of the multidisciplinary team concerning shared decision-making in treatment decisions for older patients with metastatic castration-resistant prostate cancer.	Quantitative. Cross sectional study.	A validated survey using questionaire. Descriptive statistics	(1) Elicit individual patient preferences using Decision-Aids. (2) Offer patients the opportunity to gain knowledge about their disease and values in their own time with their family.	100
Griffiths et al. (2020) [25]	Seventeen people with dementia and cancer, twenty-two relatives, and nineteen staff members (Clinical nurse specialists; *n* = 8). Recruited from Oncology and associated departments in two National Health Service (NHS) Trusts in one UK region and their local communities.	To explore cancer decision-making experiences of people with cancer and dementia, their families, and healthcare staff.	Qualitative.	Semi-structured interview. Ethnographically informed thematic analysis.	(1) Ensure people with cancer and dementia apply an individualized ability focused assessment. (2) Consider which options were appropriate for patients based on multiple factors.	100
Shen et al. (2020) [32]	Health care providers (*n* = 99), 24 advance practice providers (including nurse practitioners and physician assistants); 23 nurses, 14 social workers, 13 physicians, and 20 other health care providers. Recruited from community-based centers, cancer centers, and hospitals in the Northeastern U.S.	To evaluate a Communication Skills Training (CST) module for health care providers applying a shared decision-making approach to a meeting with an older adult with cancer and his/her family.	Quantitative. Pre/post study design.	Pre- and post-training Standardized Patient Assessments and a survey on their confidence in and intent to utilize skills taught. Descriptive statistics.	(1) Improve collaborative shared decision making among providers, patients, and family members in the context of older adults with cancer. (2) Promote an active dialogue between the triad while respecting patient values and preferences.	75
Strohschein et al. (2020) [33]	An 89-year-old man with head and neck cancer. Recruited from Cancer Center in Toronto, Canada.	To present comprehensive geriatric assessment (CGA) as an approach to personalizing care for older adults with cancer.	Qualitative. Case Study Design.	Retrospective case. describe the process of CGA and an overview of geriatric oncology screening and assessment.	(1) Integrate geriatric assessment tools into practice to identify and address age-related concerns. (2) Facilitate communication and contribute to personalization of care. (3) Spent a time on patient assessments during the decision-making process.	-
Festen et al. (2021) [26]	Two hundred fourteen patients with cancer of 70 years and older were primarily seen at the surgical outpatient clinic. Recruited from a University Medical Center, Netherlands.	A novel care pathway was set up incorporating geriatric assessment into treatment decision-making for older cancer patients. Treatment decisions could be modified following discussion in an onco-geriatric multidisciplinary team (MDT). To assess the effect of treatment modifications on outcomes.	Quantitative. Retrospective study.	Retrospective analysis of outcomes. Descriptive statistics.	(1) Incorporating nurse-led geriatric assessment in decision-making. (2) Assess the patient preferences regarding treatment outcomes. (3) Spent a time on patient assessments during the decision-making process.	100
Dijkman et al. (2022) [27]	Thirteen surgeons and thirteen nurses. Recruited from two hospitals in the northern Netherlands.	To explore how surgeons and nurses perceive the involvement of adult children of older patients with cancer in treatment decision-making.	Qualitative.	Open in-depth interviews. Thematic analysis.	(1) Ensure positive family involvement in treatment decision-making. (2) Stimulate the communication and deliberation between patients and their adult children.	100

## Data Availability

The datasets used or analyzed during the current study available from the corresponding author on reasonable request.

## Supplementary Materials

The following supporting information can be downloaded at: https://www.mdpi.com/article/10.3390/healthcare11040546/s1, Supplementary File S1. Key words; Supplementary File S2. Searches; Supplementary File S3. Scores.
