# Supplementary material for: The Role of Nurse on the Treatment Decision Support for Older People with Cancer: A Systematic Review
Source: Healthcare (Basel). 2023 Feb 12;11(4):546. doi: 10.3390/healthcare11040546 (PMC9956907; doi:10.3390/healthcare11040546)
Supplement: Supplementary file 1 [file healthcare-11-00546-s001.zip › healthcare-2187032-supplementary.pdf]

Supplementary File S1. Key words

	#1 decision making	#2 older adult	#3 role of nurse	#4 cancer
PubMed/ MeSH	Decision Making<br>Decision Support Techniques<br>Advance Care planning<br>Advance Directives	Aged (65+)<br>Aged, 80 and over<br>Frail Elderly	Nurse's Role<br>Nurses<br>Health Personnel	Neoplasms
PubMed/ related terms	"Decision Making"<br>"Decision Support"<br>"Decision Aid"<br>"Patient Centered Care"<br>"Advance Health Care Planning"<br>"Advance Medical Planning"	"Older people"<br>"Older Adult"<br>Elderly<br>"Old Person"<br>"Older Person"	"role of nurse"<br>"Nursing Role"<br>"Professional Role"<br>"Medical Personnel"<br>"Health Care Provider"<br>"Healthcare Worker"<br>"Health Care Professional"<br>"Health Care Staff"	cancer<br>Tumor<br>Malignan*
CINAHL/ Hedings	decision making+<br>Decision Support Techniques+<br>Advance Care Planning<br>Patient Centered Care<br>Advance Directives+	Aged+<br>Aged, 80 and Over+<br>Frail Elderly	Nursing Role<br>Professional Role+<br>Nurses+<br>Health Personnel+	Neoplasms+

	#1 decision making	#2 older adult	#3 role of nurse	#4 cancer
CINAHL/ related terms	Decision making<br>Decision Support<br>Decision Aid<br>Advance Health Care Planning<br>Advance Medical Planning	Older people<br>Older Adult<br>Elderly<br>Old Person<br>Older Person	role of nurse<br>Nurse's Role<br>Health Care Staff<br>Medical Personnel<br>Health Care Provider<br>Healthcare Worker<br>Health Care Professional	cancer<br>Tumor<br>Malignan*
PsycInfo/ Index Terms	decision making<br>Patient Centered Care<br>Advance Directives		Professional Role+<br>Nurses+<br>Health Personnel+<br>Medical Personnel+	Neoplasms+
PsycInfo/ related terms	Decision making<br>Decision Support<br>Decision Aid<br>Advance Care Planning<br>Advance Health Care Planning<br>Advance Medical Planning	aged<br>Older people<br>Older Adult<br>Elderly<br>Old Person<br>Older Person	role of nurse<br>Nurse's Role<br>Nursing Role<br>Health Care Provider<br>Healthcare Worker<br>Health Care Professional<br>Health Care Staff	cancer<br>Tumor<br>Malignan*

Supplementary File S2.　Searches

PubMed

Search number	Query	Results
1	"Decision Making"[MeSH Terms] OR "decision support techniques"[MeSH Terms] OR "advance care planning"[MeSH Terms] OR "advance directives"[MeSH Terms] OR "Decision Making"[All Fields] OR "Decision Support"[All Fields] OR "Decision Aid"[All Fields] OR "Patient Centered Care"[All Fields] OR "Advance Health Care Planning"[All Fields] OR "Advance Medical Planning"[All Fields]	499,604
2	"aged"[MeSH Terms] OR "aged, 80 and over"[MeSH Terms] OR "frail elderly"[MeSH Terms] OR "Older people"[All Fields] OR "Older Adult"[All Fields] OR "aged"[MeSH Terms] OR "aged"[All Fields] OR "elderly"[All Fields] OR "elderlies"[All Fields] OR "elderly s"[All Fields] OR "elderlys"[All Fields] OR "Old Person"[All Fields] OR "Older Person"[All Fields]	5,937,341
3	"nurse s role"[MeSH Terms] OR "nurses"[MeSH Terms] OR "health personnel"[MeSH Terms] OR "role of nurse"[All Fields] OR "Nursing Role"[All Fields] OR "Professional Role"[All Fields] OR "Medical Personnel"[All Fields] OR "Health Care Provider"[All Fields] OR "Healthcare Worker"[All Fields] OR "Health Care Professional"[All Fields] OR "Health Care Staff"[All Fields]	641,597
4	"neoplasms"[MeSH Terms] OR "cancer s"[All Fields] OR "cancerated"[All Fields] OR "canceration"[All Fields] OR "cancerization"[All Fields] OR "cancerized"[All Fields] OR "cancerous"[All Fields] OR "neoplasms"[MeSH Terms] OR "neoplasms"[All Fields] OR "cancer"[All Fields] OR "cancers"[All Fields] OR "cysts"[MeSH Terms] OR "cysts"[All Fields] OR "cyst"[All Fields] OR "neurofibroma"[MeSH Terms] OR "neurofibroma"[All Fields] OR "neurofibromas"[All Fields] OR "tumor s"[All Fields] OR "tumoral"[All Fields] OR "tumorous"[All Fields] OR "tumour"[All Fields] OR "neoplasms"[MeSH Terms] OR "neoplasms"[All Fields] OR "tumor"[All Fields] OR "tumour s"[All Fields] OR "tumoural"[All Fields] OR "tumourous"[All Fields] OR "tumours"[All Fields] OR "tumors"[All Fields] OR "malignan*"[All Fields]	5,332,532
	#1 AND #2 AND #3 AND #4	1,656

CINAHL　※「+」 narrower terms

Search number	Query	Results

S1	(MH "Decision Making+") OR (MH "Decision Support Techniques+") OR (MH "Advance Care Planning") OR (MH "Patient Centered Care") OR (MH "Advance Directives+") OR Decision making OR Decision Support OR Decision Aid OR Advance Health Care Planning OR Advance Medical Planning	227,782
S2	(MH "Aged+") OR (MH "Aged, 80 and Over+") OR (MH "Frail Elderly") OR Older people OR Older Adult OR Elderly OR Old Person OR Older Person	976,381
S3	(MH "Nursing Role") OR (MH "Professional Role+") OR (MH "Nurses+") OR (MH "Health Personnel+") OR role of nurse OR Nurse's Role OR Health Care Staff OR Medical Personnel OR Health Care Provider OR Healthcare Worker OR Health Care Professional	773,074
S4	(MH "Neoplasms+") OR cancer OR Tumor OR Malignan*	61,839
	S1 AND S2 AND S3 AND S4	856

APA PsycInfo ※blue words are narrower terms

Search number	Query	Results
S1	((DE "Decision Making" OR DE "Choice Behavior" OR DE "Group Decision Making" OR DE "Management Decision Making") OR (DE "Patient Centered Care")) OR (DE "Advance Directives") OR decision making process OR Decision Support OR Decision Aid OR Advance Care Planning OR Advance Health Care Planning OR Advance Medical Planning	141,500
S2	aged OR Older people OR Older Adult OR Elderly OR Old Person OR Older Person	707,574

S3	(((DE "Professional Role" OR DE "Counselor Role" OR DE "Professional Boundaries" OR DE "Therapist Role") OR (DE "Nurses" OR DE "Psychiatric Nurses" OR DE "Public Health Service Nurses" OR DE "School Nurses")) OR (DE "Health Personnel" OR DE "Allied Health Personnel" OR DE "Caregivers" OR DE "Medical Personnel" OR DE "Mental Health Personnel")) OR (DE "Medical Personnel" OR DE "Dentists" OR DE "Military Medical Personnel" OR DE "Nurses" OR DE "Optometrists" OR DE "Pharmacists" OR DE "Physical Therapists" OR DE "Physicians" OR DE "Psychiatric Hospital Staff") OR role of nurse OR Nurse's Role OR Nursing Role OR Health Care Provider OR Healthcare Worker OR Health Care Professional OR Health Care Staff	179,049
S4	(DE "Neoplasms" OR DE "Benign Neoplasms" OR DE "Breast Neoplasms" OR DE "Endocrine Neoplasms" OR DE "Leukemias" OR DE "Melanoma" OR DE "Metastasis" OR DE "Nervous System Neoplasms" OR DE "Terminal Cancer") OR cancer OR Tumor OR Malignan*	114,721
	S1 AND S2 AND S3 AND S4	491

Supplementary File S3. Scores

**Reference**	**S1. Are there clear research questions?**	**S2. Do the collected data allow to address the research questions?**	**1. Qualitative**	1.1. Is the qualitative approach appropriate to answer the research question?	1.2. Are the qualitative data collection methods adequate to address the research question?	1.3. Are the findings adequately derived from the data?	1.4. Is the interpretation of results sufficiently substantiated by data?	1.5. Is there coherence between qualitative data sources, collection, analysis and interpretation
			**2. Quantitative randomized controlled trials**	2.1. Is randomization appropriately performed	2.2. Are the groups comparable at baseline?	2.3. Are there complete outcome data?	2.4. Are outcome assessors blinded to the intervention provided?	2.5 Did the participants adhere to the assigned intervention?
			**3. Quantitative non-randomized**	3.1. Are the participants representative of the target population?	3.2. Are measurements appropriate regarding both the outcome and intervention (or exposure)?	3.3. Are there complete outcome data?	3.4. Are the confounders accounted for in the design and analysis?	3.5. During the study period, is the intervention administered (or exposure occurred) as intended?
			**4. Quantitative descriptive**	4.1. Is the sampling strategy relevant to address the research question?	4.2. Is the sample representative of the target population?	4.3. Are the measurements appropriate?	4.4. Is the risk of nonresponse bias low?	4.5. Is the statistical analysis appropriate to answer the research question?
			**5. Mixed methods**	5.1. Is there an adequate rationale for using a mixed methods design to address the research question	5.2. Are the different components of the study effectively integrated to answer the research question?	5.3. Are the outputs of the integration of qualitative and quantitative components adequately interpreted	5.4. Are divergences and inconsistencies between quantitative and qualitative results adequately addressed	5.5. Do the different components of the study adhere to the quality criteria of each tradition of the methods involved?
Tariman et al., 2014	YES	YES	5	YES	YES	YES	YES	YES

Bridges et al., 2015	YES	YES	1	YES	YES	YES	YES	YES
Burton et al., 2017	YES	YES	4	YES	YES	YES	YES	YES
Jones, et al., 2018	YES	YES	1	YES	YES	YES	YES	YES
McWilliams et al., 2018	YES	YES	1	YES	YES	YES	YES	YES
Sattar et al., 2018	YES	YES	1	YES	YES	YES	YES	YES
de Angst et al., 2019	YES	YES	4	YES	YES	YES	YES	YES
Griffiths et al., 2020	YES	YES	1	YES	YES	YES	YES	YES
Shen et al., 2020	YES	YES	3	YES	YES	YES	NO	YES
Festen et al., 2021	YES	YES	4	YES	YES	YES	YES	YES
Dijkman et al., 2022	YES	YES	1	YES	YES	YES	YES	YES
